# 5-Alpha Reductase Inhibitors on Hypersexuality During the Manic Phase of Bipolar and Psychotic Patients; New Insight to a Well-Known Medicines

**DOI:** 10.5812/ijpr-156707

**Published:** 2025-02-09

**Authors:** Mitra Mahmoudi Meymand, Saeed Mohammad Soleymani, Hadi Esmaily

**Affiliations:** 1 Department of Clinical Pharmacy, School of Pharmacy, Shahid Beheshti University of Medical Sciences, Tehran, Iran; 2 Clinical Research Development Centre, Imam Hossein Educational Hospital, Shahid Beheshti University of Medical Sciences, Tehran, Iran

**Keywords:** Finasteride, Dutasteride, 5-alpha Reductase (5AR), Hypersexuality, Bipolar Disorder, Schizoaffective Patients

*Dear Editor,*

Bipolar disorder (BD) is a complex mental health condition characterized by dramatic shifts in mood and energy that can be so pronounced they disrupt an individual's ability to manage daily tasks and make even routine activities feel overwhelming (1). One of the early symptoms of a manic episode of BD is hypersexuality (2). Individuals affected by this condition often experience a heightened sex drive that goes far beyond societal expectations, which can lead to impulsive or even risky sexual behavior (2). The exact cause of hypersexuality in BD is not fully understood, but it is likely related to changes in brain chemistry and the reward pathways activated during manic episodes. The surge of mood-enhancing neurotransmitters like dopamine, endorphins, and oxytocin creates a strong sense of pleasure, which may drive the patient to seek sexual arousal (3). Hypersexuality during the manic phase can result from hormone imbalance and the interaction between estrogen and testosterone (3).

Schizophrenia is a multifactorial disorder in which genetics and environmental factors play an important role in its pathogenesis. The most important neurotransmitters in the pathogenesis of this disorder are dopamine, glutamate, and serotonin. Additionally, sex hormones, including testosterone, play an important role in sexual characteristics and affect brain development (4). Testosterone plays a direct and indirect role in modulating the mesocorticolimbic system. By acting on tyrosine hydroxylase in the ventral tegmental area, which contains androgen receptors, testosterone increases the density of dopaminergic neurons in this area by increasing the synthesis of dopamine and inhibiting its reabsorption (5).

5-alpha reductase (5AR) inhibitors, by reducing the level of dihydrotestosterone (DHT), have a significant effect on reducing dopamine (D1 and D3 receptors) and the synthesis of neurosteroids in this area (6, 7). The levels of testosterone and its precursor dehydroepiandrosterone (DHEA) are increased in schizophrenic patients. Testosterone and its precursors are increased in patients with the first episode of psychosis, relapse periods of schizophrenia, and women with several episodes of stable psychosis. The high level of testosterone in men compared to women is probably one of the factors justifying the increased prevalence of psychosis in men compared to women (8). The neurotransmitters dopamine, glutamate, and GABA, which are partly responsible for the primary mechanisms of schizophrenia, are affected by DHEA and testosterone (9).

Finasteride, a selective inhibitor of type II 5AR enzyme, inhibits the conversion of testosterone to DHT and is approved for the treatment of benign prostate hyperplasia (BPH) and androgenetic alopecia. It decreases DHT concentrations in serum and scalp by up to 70% and 60%, respectively (10). Dutasteride inhibits both types of 5α-reductases irreversibly. Type II is responsible for about 66% of circulating DHT. The dual inhibition of type I and II leads to a near-complete suppression of DHT (11).

We aim to hypothesize the potential role of 5AR inhibitors as a treatment option for hypersexuality during manic episodes in bipolar and schizoaffective disorders. Currently, there are no published clinical trials investigating the effectiveness of finasteride or dutasteride in these cases. However, four published studies, including a case report, a case series involving 11 male patients, a case series involving 10 male patients, and an animal study, have provided some evidence of the beneficial effects of finasteride in psychiatric disorders.

The first evidence, reported by Wang and Lin, is a case report of an 86-year-old man with benign prostatic hyperplasia (BPH) who was successfully treated with finasteride for inappropriate sexual behavior refractory to psychotropic medications (12). The second is a letter to the editor regarding 11 men who received 5 mg of finasteride once daily for 12 weeks, showing that inappropriate sexual behaviors disappeared in six of the patients after 8 weeks of treatment. Two of these patients experienced a return of symptoms, which improved after continuing finasteride for an additional 4 weeks (13). These studies also suggested finasteride as a new therapeutic option for schizophrenia and other neuropsychiatric disorders (14).

A report published as a poster presentation in 2008 showed an improvement in voiding symptoms in 10 men with vascular dementia a few months after starting finasteride treatment. All enrolled patients experienced a reduction in hypersexuality, with no reported adverse effects, effectively reducing libido and hypersexual behavior in patients (15). Neurotransmitters like dopamine and serotonin affect sexual behavior (14). Another mechanism by which finasteride reduces hypersexuality is by decreasing the serum DHT concentration, the only independent hormonal predictor of orgasm frequency (16).

An animal study conducted in 2017 showed that finasteride at doses of 25 and 50 mg/kg significantly inhibited motor behaviors in adolescent rats (P < 0.01 and P < 0.05, respectively). The percentage decrease ranged from 31% to 68% in exploratory behaviors and from 33% to 66% in motor behaviors. Finasteride inhibited motor behaviors more effectively at higher doses. The reduction in open-field behaviors is attributed to the inhibition of testosterone conversion to DHT. Additionally, the study reported a decrease in dopamine and its metabolites in the frontal cortex, hippocampus, caudate putamen, and nucleus accumbens (17).

There are no studies on the use of dutasteride for the treatment of psychiatric disorders or hypersexuality. However, in a clinical trial involving 117 men with androgenic alopecia, the incidence of sexual adverse events was nearly twice as high in the group receiving dutasteride at a dose of 0.5 mg over 24 weeks. This increase is attributed to the potent inhibition of DHT (18).

**Figure 1.**
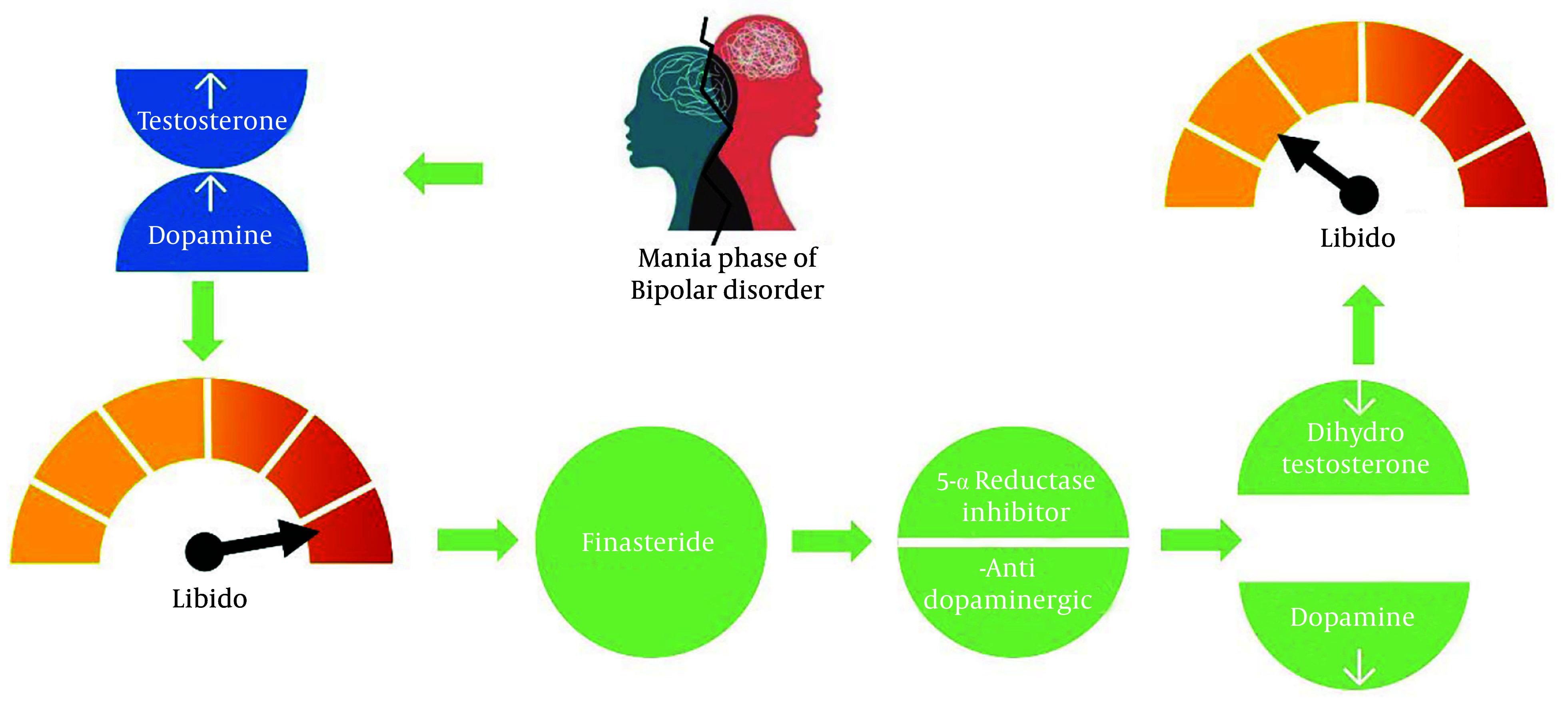
Schematic of the possible mechanisms of 5-alpha reductase (5AR) inhibitors in hypersexuality during the manic phase of bipolar and psychotic patients

Due to multifactorial mechanisms—both modulating neurotransmitters that affect neural excitability and decreasing levels of dopamine and DHT (Figure 1) —initial findings are promising. However, controlled trials are required. Finasteride is a well-known medication with an acceptable adverse effect profile, making it a desirable option. Nevertheless, its long-term impact on neurotransmitter levels in patients with BD remains unknown, and factors such as baseline neurotransmitter levels and coexisting conditions need to be considered. Clinicians must carefully weigh the potential benefits against the risks, especially in women of childbearing age, due to associated safety concerns.
